# Juvenile growth and survival of the apple snail *Pomacea canaliculata* (Caenogastropoda: Ampullariidae) reared at different constant temperatures

**DOI:** 10.1186/2193-1801-2-312

**Published:** 2013-07-11

**Authors:** María E Seuffert, Pablo R Martín

**Affiliations:** Departamento de Biología, Bioquímica y Farmacia, Universidad Nacional del Sur, San Juan 670, Bahía Blanca, 8000 Argentina; Consejo Nacional de Investigaciones Científicas y Técnicas (CONICET), Av. Rivadavia 1917, Ciudad Autónoma de Buenos Aires, C1033AAJ Argentina

## Abstract

**Background:**

*Pomacea canaliculata* is a freshwater snail that cultured under certain conditions could provide interesting rewards in research and aquaculture. *P*. *canaliculata* is usually reared at 25°C, though the optimal temperature for culturing this species, that balances growth and survival rates, is so far unknown. In this work we present results of growth and survival of cohorts reared in the laboratory at different constant water temperatures (15, 20, 25, 30 and 35°C) during the pre-reproductive period.

**Findings:**

Two different groups were recognized among the five treatments: the two lower temperatures (15 and 20°C) that showed no mortality but with very low growth rates and the treatments of 25, 30 and 35°C in which snails grew faster but displayed a reduction in survival as temperature increases. After 10 weeks, the mean shell lengths attained at 30 and 35°C were only 2–3 mm higher than that of the treatment of 25°C and were not statistically different.

**Conclusions:**

Our results support using water temperatures of 25°C for the rearing of cohorts when the objective is to quickly obtain numerous large snails. Temperatures of 15 and 20°C may be appropriate if the aim is to preserve juveniles for long periods with a very low risk of mortality. The results reported here will be useful to the scheduling of laboratory trials intended for basic research, snail control or mass rearing for different applications of this species.

## Background

*Pomacea canaliculata* (Lamarck) is a freshwater snail belonging to the Ampullariidae family, native to subtropical and temperate South America. It naturally distributes from southern Brazil to the south of Buenos Aires Province, where it reaches its southernmost limit in Encadenadas del Oeste basin (Martín et al. 2001). The interest on the basic aspects of the biology and ecology of this species has greatly increased in the past years due to the serious troubles caused to crops and natural wetlands in invaded regions, especially in Southern and Eastern Asia. The introduction of *P*. *canaliculata* and other apple snails to new areas needs to be strongly discouraged due to their negative impacts and great invasive potential (Cowie et al. 2009; EFSA 2012). Nevertheless, in their native ranges, in already invaded areas or under rigorously confined conditions, their culture promises interesting rewards in the fields of applied and basic research and aquaculture.

Several studies were intended to adjust the culturing techniques of different species of ampullariids, such as *Pomacea patula catemacensis* (Baker) (Ruiz-Ramírez et al. 2005), *Pomacea paludosa* (Say) (Garr et al. 2011; Posch et al. 2012), *Pomacea bridgesii* (Reeve) (Coelho et al. 2012), *Pomacea urceus* (Müller) (Ramnarine 2003,2004) and *Marisa cornuarietis* L. (Aufderheide et al. 2006; Selck et al. 2006). There are some researches in which *P*. *canaliculata* cohorts were reared under laboratory conditions, both at room temperature (9-29°C; Estebenet and Cazzaniga 1992) and with controlled temperature (Estebenet and Cazzaniga 1992; Estoy et al. 2002; Martín and Estebenet 2002; Tamburi and Martín 2009). However, the latter were all performed at 25°C, thus there is so far no studies of the response of growth and survival of *P*. *canaliculata* to a wide temperature range.

It is widely recognized that several aspects of the life cycle of *P*. *canaliculata*, including growth, feeding, crawling, aerial respiration, reproduction and survival, depend on temperature (*e*.*g*. Estebenet and Cazzaniga 1992; Estebenet and Martín 2002; Ito 2002; Albrecht et al. 2004; Matsukura and Wada 2007; Seuffert and Martín 2009; Seuffert et al. 2010). For instance, in short duration trials the levels of general activity and feeding reached a peak in the range of 25-32°C (Heiler et al. 2008; Seuffert et al. 2010) but began to decrease above these temperatures with an associated declined of survival (Seuffert and Martín 2010). On the other hand, the activity of *P*. *canaliculata* drops rapidly to zero below 15°C (Seuffert et al. 2010). Based on the response of these traits, we hypothesized that temperatures higher than 25°C will probably promote a faster growth though also will reduce survival. The optimal temperature for culturing this species, that maximizes growth and survival rates, is so far unknown although it also depends on the specific aims of the culture. In this work we present results of growth rates and survival of *P*. canaliculata reared at different constant water temperatures during the pre-reproductive period (ten weeks).

## Methods

Ten egg masses were collected in February 9^th^ 2012 in El Huascar stream, (36°55′3″ S, 61°35′50″W, Buenos Aires Province, Argentina) and brought to the laboratory where they were kept in a room at 25°C. Most eggs hatched between February 14^th^ and 22^nd^ and the hatchlings from each egg mass were raised for 3–4 weeks in 3 L aquaria. Afterwards, a group of 120 snails was randomly selected (shell length = 5.00 ± 0.046 mm; mean ± standard error) and subgroups of 12 individuals were placed in ten plastic aquaria of 20 L (30 × 35 × 20 cm) that were maintained at five constant water temperatures with electric thermostats (15, 20, 25, 30 and 35°C; two aquaria for each temperature) under 14 h light/day photoperiod. Once a week the aquaria were brushed, the water was totally renewed and snails’ shell length (SL) was measured with a Vernier caliper to the nearest 0.1 mm; the number of live snails per aquarium was also recorded. Throughout the whole trial the snails were fed with fresh lettuce which was maintained at *ad libitum* levels by supplying it two to three times a week according to consume, in order to avoid bacterial growth and water fouling. No artificial aeration was provided as it affects water quality by resuspending food debris and faeces and since the aquarium dimensions and shape allowed the snails an easy access to breathe air.

For each temperature we calculated the growth rate (mm.week^-1^) for the phase of linear growth (first four weeks), the mean shell length after four and ten weeks, the percentage of survival (100 × number of live snails / total number of snails) at the end of the experiment and the mean survival time. Differences in these variables among treatments were analyzed with nested ANOVAs, with the aquaria being the nested factor and water temperature the main fixed factor. When homogeneity of variances was rejected by Levene’s test, the dependent variable was log-transformed and re-analyzed. If homoscedasticity was not attained by this procedure, a non-parametric test (Kruskal-Wallis) was performed. Differences among treatments in the coefficients of variation (CV,%) of shell length were compared through Levene’s tests using the individual deviations relative to the median of each treatment (Schultz 1985).

## Results and discussion

**Figure 1:**
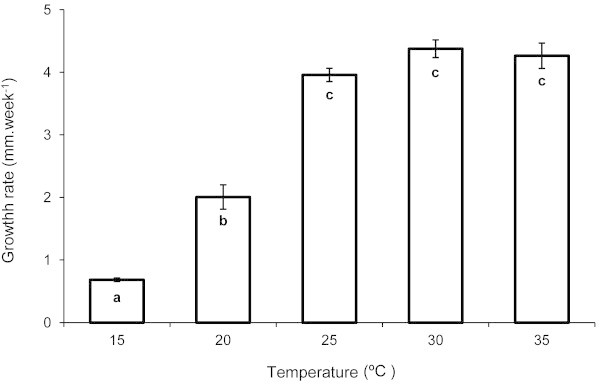
**Growth rates of*Pomacea canaliculata*at five constant water temperatures.** Growth rates of *Pomacea canaliculata* (mean ± SE) for the first four weeks of rearing at five constant water temperatures; different letters indicate significantly different mean shell lengths at week four.

**Figure 2:**
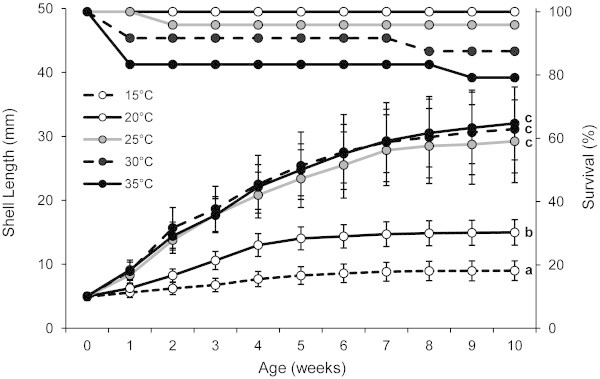
**Percentage of survival and shell length of*Pomacea canaliculata*at five constant water temperatures.** Percentage of survival and shell length (mean ± SD) of *Pomacea canaliculata* reared during ten weeks at five constant water temperatures; different letters indicate significantly different shell lengths at week ten.

The growth rate during the first four weeks varied 6.4 fold in the studied temperature range, between a minimum of 0.684 mm.week^-1^ at 15°C and a maximum of 4.377 mm.week^-1^ at 30°C (Figure 1). The values estimated for the treatments of 25 and 35°C were 3.958 and 4.264 mm.week^-1^, respectively. The sizes of the snails after four weeks were significantly different among treatments (F_*4*,*5*_ = 111.670; *p* < 0.0001), being higher for the treatments of 25, 30 and 35°C (t-tests; *p* < 0.0001 in all cases, after Bonferroni correction for 10 comparisons; Figures 1 and 2); there was no significant component of variance due to the different aquaria (nested factor; F_*5*,*102*_ = 1.458; *p* = 0.210).

After ten weeks, the maximum mean shell length (32.03 mm) was recorded in the treatment of 35°C (Figure 2), being significantly higher than the means recorded with 15 and 20°C (*p* < 0.0001 in both cases, after Bonferroni correction) but not different from those of 25°C (*p* = 0.078) and 30°C (*p* = 0.577). The mean shell lengths for the treatments of 25 and 30°C were 29.24 and 31.14 mm, respectively, and were not significantly different (*p* = 0.216). Mean survival time differed significantly among treatments (Kruskal-Wallis test: Χ^2^ = 9.639; *p* = 0.047) and was negatively correlated with water temperature (Spearman’s ρ = −0.279; *p* = 0.002). The percentages of survival after ten weeks for the treatments of 15 and 20°C were 100%, whereas for 25, 30 and 35°C values were 95.83, 87.50 and 79.17%, respectively (Figure 2).

On the whole, two different groups were clearly recognized among the five treatments: the two lower temperatures (15 and 20°C) that showed no mortality and very low growth rates and, on the other side, the treatments of 25, 30 and 35°C in which snails grew faster but suffered some mortality. Within the latter group of temperatures, growth rate at 30°C was higher than at 25°C, though the differences were relatively small (0.422 mm.week^-1^) and not statistically different; also, both mean shell lengths were not different. A higher temperature (35°C) did not increase the growth rate or the mean shell length after the first four weeks. After 10 weeks, the mean lengths attained with 30 and 35°C were only 2–3 mm higher than that of the treatment of 25°C and were not statistically different. These results, together with the lower survival registered with the two warmer temperatures, indicates that there would be no apparent benefit in culturing snails of this species at temperatures of 30°C and above. On the other hand, no snail died after 10 weeks in the treatments of 15 and 20°C though the slow growth recorded with these low temperatures would imply an undesirable long period of rearing.

The lack of a further increase in growth rate in the treatments of highest temperatures was probably related to a decrease in the time spent feeding above 25°C (Seuffert et al. 2010). Similarly, the weight growth rate of *Pomacea maculata* at 35°C was smaller than that between 20 and 30°C, even though in this case the feeding rate was not affected by water temperature (Gettys et al. 2008).

As time passed, the differences in size among snails of the same treatment got bigger. This was reflected in the increase of the coefficient of variation (CV) of shell length (Table 1). At the beginning of the experiment, the CV was not different among treatments (Levene’s test: F_*4*,*115*_ = 0.576, *p* = 0.681; mean CV: 11.25%, range: 10.3-12.3%), but at week ten it reached values that ranged from 13.3% at 20°C to 22.0% at 25 and 30°C and that were significantly different (Levene’s test: F_*4*,*105*_ = 3.365, *p* = 0.012). Size is sexually dimorphic in *P*. *canaliculata* (Estebenet and Martín 2003) but this probably had no influence in this size variation since none of the snails attained maturity during our ten weeks trial; under optimum conditions males and females take at least 13 weeks to mature (Tamburi and Martín 2009). A possible explanation of the differences in size recorded here is the feeding interference between individuals (Estebenet and Martín 2002). If the aim is to obtain homogeneous stocks of snails for experimentation this can be solved by using individual aquaria (Tamburi and Martín 2009) or individual plastic frame enclosures in collective aquaria (Estebenet and Cazzaniga 1998) or by rearing them at low temperatures (15-20°C).

**Table 1:** **Coefficients of variation of shell length of*Pomacea canaliculata*at five constant water temperatures**

	15°C	20°C	25°C	30°C	35°C
Week 0	12.3	11.5	11.3	10.9	10.3
Week 10	17.0	13.3	22.1	22.0	17.8

Coefficients of variation (CV,%) of shell length of *Pomacea canaliculata* reared during ten weeks at five constant water temperatures; CVs were calculated at the beginning (week 0) and at the end (week 10) of the experiment.

In the present work we found that, as expected, survival and growth showed opposite responses to temperature which indicates that the optimal development of laboratory cohorts of *P*. *canaliculata* would occur at intermediate values. Until now, rearing temperatures close to 25°C have been frequently chosen for the design of laboratory experiments (e.g., Estebenet and Cazzaniga 1992; Estoy et al. 2002; Martín and Estebenet 2002; Tamburi and Martín 2009). Our results support using water temperatures of 25°C for the rearing of cohorts when the objective is to quickly obtain numerous large snails. On the other hand, temperatures of 15 and 20°C may be appropriate if the aim is to preserve juveniles for long periods with a very low risk of mortality.

The results reported here will be useful to improve the culturing techniques of *P*. *canaliculata* that will help to promote different applications of this species, such as its use as a component of high nitrate content in biological fertilizers (Vetayasuporn 2006) or as food for aquaculture (Bombeo-Tuburan et al. 1995; Lan et al. 2006) and for intensive animal farming (Kaensombath and Ogle 2005). The information obtained here will be also useful to the scheduling of laboratory trials intended for basic research, snail control (Joshi et al. 2008; Song et al. 2011) or mass rearing for ecotoxicological applications (Lo and Hsieh 2000; Coler et al. 2005; Vega et al. 2012).

*Pomacea canaliculata* is the apple snail that reaches the most extreme latitudes in the world, both in its native range (Martín et al. 2001) and in invaded regions (Ito 2002). In consequence, its response to low temperatures may differ from that of subtropical and tropical *Pomacea* spp., indicating that is necessary to be cautious in extrapolating the present results to them and also to obtain data on thermal biology at the species or population level.
